# Clinical response to FGFR inhibitors in patients with FGFR2-amplified advanced gastric cancer: two case reports

**DOI:** 10.3389/fonc.2026.1828167

**Published:** 2026-07-07

**Authors:** Jing-Jing Li, Yu- Qian, Hui-Ting Xu, Yan-Li Nie

**Affiliations:** Department of Medical Oncology, Hubei Cancer Hospital, Tongji Medical College, Huazhong University of Science and Technology, Wuhan, Hubei, China

**Keywords:** adverse events, FGFR2, gastric cancer, infigratinib, pemigatinib, response evaluation

## Abstract

Patients with gastric cancer (GC) harboring fibroblast growth factor receptor 2 (FGFR2) amplification showed significantly worse survival, compared with patients with FGFR2-unamplified GC. Although various targeted drugs have been developed, previous studies have shown that their efficacy remains suboptimal in most patients within this subgroup. We retrospectively present two cases of FGFR2-amplified advanced GC treated with FGFR inhibitors. Case 1 was a 52-year-old female diagnosed with stage IVB (cTxN+M1, bone and lymph node metastases, HER2-negative, CPS = 2) poorly differentiated signet-ring cell carcinoma, harboring high-level FGFR2 amplification (gene copy number=24.7). Due to extensive bone metastases resulting in severe anemia, pain, and limited mobility (performance status 3), the patient was intolerant to chemotherapy and immunotherapy. In the first-line setting, she received pemigatinib, achieving a progression-free survival (PFS) of 4.4 months and an overall survival (OS) of 8.3 months, accompanied by a significant decrease in tumor markers and rapid clinical symptom improvement. Case 2 was a 61-year-old female diagnosed with stage IVB (cT4bN+M1, liver and lymph node metastases, HER2-negative, CPS = 5) poorly differentiated adenocarcinoma. In the third-line setting, she received infigratinib, attaining a PFS of 5.7 months and an OS of 18.5 months. Both patients experienced predictable and manageable FGFR inhibitor-related adverse events (e.g., keratitis, onychotoxicity, hyperphosphatemia). This case series offers hypothesis-generating observations suggesting potential activity of FGFR inhibitors (such as pemigatinib and infigratinib) in patients with FGFR2-amplified advanced gastric cancer who have limited conventional therapeutic options or are intolerant to chemotherapy, highlighting the importance of next-generation sequencing-guided precision therapy.

## Introduction

Gastric cancer (GC) is the fifth leading cause of cancer-related mortality worldwide and the fifth most prevalent malignancy (1). In 2022, there were over 968,000 new cases of GC and close to 660,000 deaths, with incidence and mortality rates accounting for 4.9% and 6.8%, respectively. Owing to the insidious nature of early gastric cancer symptoms, most patients are diagnosed at advanced stages where therapeutic options are limited and outcomes suboptimal, with a median overall survival typically under one year (2, 3). Surgical resection is the mainstay treatment for gastroesophageal junction (GEJ) cancer, whereas cytotoxic chemotherapy remains the backbone for treating metastatic gastric cancer. Few treatment options exist after the failure of standard therapy (4, 5). Targeted therapies and immunotherapeutic agents have improved survival for patients with advanced GC (6). However, the management of GC remains a formidable challenge, highlighting the critical need for novel therapeutic targets.

In recent years, fibroblast growth factor receptors (FGFRs) have emerged as key factors involved in tumorigenesis, remodeling the tumor microenvironment, and acquiring resistance to targeted therapies (7, 8). As a member of the receptor tyrosine kinase (RTK) family, FGFR comprises four subtypes (FGFR1-4) and 22 known ligands. Ligand binding induces FGFR dimerization and activation, triggering downstream signaling cascades including Ras/Raf/MAPK, PI3K/AKT, PLCγ, and STAT pathways, which are involved in the regulatory mechanisms of the cellular and environmental contexts (9). Activation of FGFR signaling can be triggered by gene amplification, mutation activation, and chromosomal translocation/fusion (10). In a retrospective analysis of sequencing data from 5557 solid tumors, FGFR1–4 aberrations occurred in 12.2% of gastric cancer cases, where amplifications were most prevalent (9%), followed by rearrangements (3%) and mutations (3%). And the most frequent alterations were detected in the FGFR2 gene (8%), followed by FGFR1 (3.5%), and to a lesser extent, FGFR3 and FGFR4 genes (<1%) (11). Clinical data have shown that FGFR2 amplification is significantly associated with lymph node metastasis, poorly differentiated or undifferentiated adenocarcinoma, and significantly worse survival in patients with gastric cancer (12).

For the moment, a series of molecularly targeted drugs has been developed to inhibit FGFR signaling. Based on their mechanism of action, they have been classified into three distinct categories: small molecule FGFR tyrosine kinase inhibitors (TKIs), anti-FGFR antibodies, and FGF ligand traps (10). Small molecule TKIs currently represent the most widespread therapeutic cancer approach, and several FGFR inhibitors are already in clinical practice: erdafitinib, pemigatinib, infigratinib, and futibatinib (13, 14). Previous research has established FGFR as a pivotal target in treating cancers such as cholangiocarcinoma and urothelial carcinoma. Similarly, research targeting FGFR in gastric cancer is rapidly expanding, and corresponding targeted therapies are emerging as important new options for gastric cancer patients (15, 16).

Here, we report two cases of FGFR2-amplified advanced gastric cancer identified by next-generation sequencing (NGS) and treated with FGFR inhibitors, including first-line pemigatinib in a chemotherapy-intolerant patient, third-line infigratinib with limited options, and sequential response to futibatinib after pemigatinib progression, providing hypothesis-generating observations suggesting potential activity and highlighting the importance of NGS-guided precision therapy. The reporting of this study conforms to the CARE guidelines.

## Case presentation

### Case report 1

**Figure 1:**
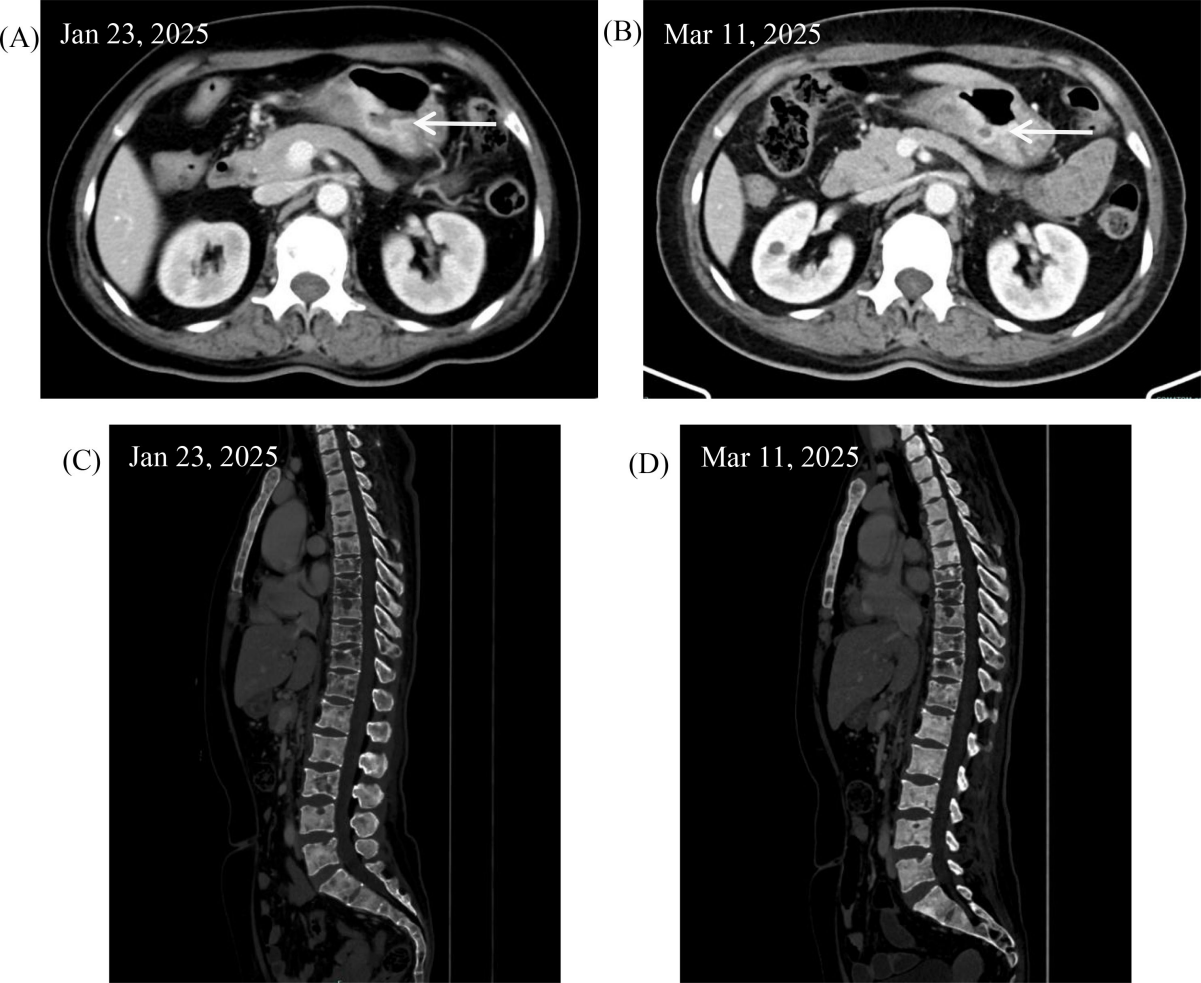
Follow-up CT scans indicating a stable disease (SD) after two and four cycles of first-line pemigatinib therapy. **(A, B)** Case 1 showing regression of gastric ulcerative lesions. **(C, D)** The lesion developed partial sclerotic fill-in of lytic lesions.

**Figure 2:**
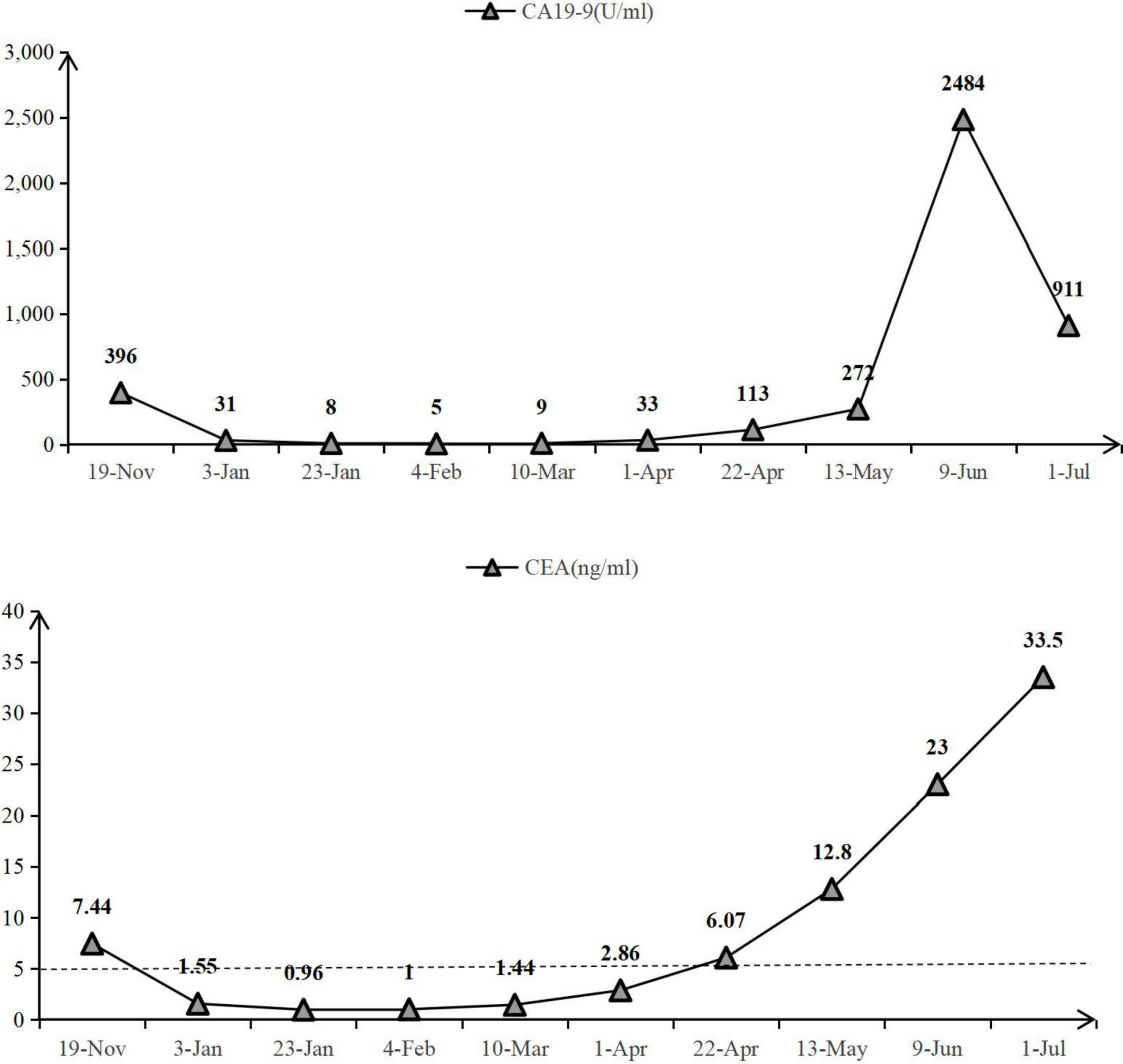
Changes in CA19–9 and CEA levels of case 1.

A 52-year-old woman with no significant past medical or family history was diagnosed with gastric carcinoma in November 2024 [cStage IVB, cTxN+M1 (bone and lymph nodes metastases), poorly differentiated signet-ring cell carcinoma, HER2-negative, CPS = 2, microsatellite stable]. Computed tomography (CT) revealed extensive bone metastases, including the left humerus, sternum, bilateral scapulae and ribs, cervical, thoracic, lumbar, and sacral vertebrae, pelvic bones, and bilateral femora. The resulting severe anemia, significant backache, and functional impairment (performance status 3) rendered her intolerant to systemic chemotherapy and immunotherapy. Stereotactic body radiation therapy (SBRT) to the lumbar vertebrae, delivering a dose of 18 Gy in 3 fractions, was administered to prevent spinal cord compression and alleviate pain. Subsequent NGS using the Burning Rock platform (a 520-gene panel) on the gastric biopsy specimen, with sequencing performed on an Illumina platform, identified high-level FGFR2 amplification (copy number 24.7) together with co-existing alterations including GRIN2A exon 11 missense (c.2146G>A, VAF 9.36%), GRM3 exon 4 missense (c.2078T>A, VAF 2.66%), and TP53 exon 6 splice region mutation (c.560-3T>G, VAF 13.25%). With informed consent for off-label use and approval from the hospital’s Cancer Genomics Medical Committee, first-line therapy with pemigatinib (inhibitors of FGFR1, 2, and 3) (13.5 mg once daily, days 1-14, 21-day cycles) was initiated. After the first cycle, her anemia resolved, analgesic use decreased, and performance status (PS) improved to 2. Tumor markers (CEA and CA19-9) declined markedly. To enhance efficacy, S-1 was added starting from cycle 2 (40 mg twice daily, days 1-14, 21-day cycles), later escalated to 60 mg twice daily at cycle 3. After five cycles of combination therapy, contrast-enhanced CT (target lesions: primary gastric lesion and retroperitoneal lymph nodes) was performed to assess response according to RECIST version 1.1. The sum of the diameters of the target lesions decreased by 2%, indicating stable disease (SD) as shown in **Figure 1**, and sclerosis of bone metastatic lesions was observed in **Figure 2**. This was accompanied by sustained decreases in tumor markers and improved performance status.

However, disease progression (24% increase) occurred after completion of five cycles of pemigatinib combined with S-1 therapy. Given the patient’s rapidly progressing disease and extremely poor prognosis, subsequent treatment regimens were adjusted frequently. Following initial first-line therapy with pemigatinib, the patient actively received chemotherapy containing oxaliplatin (administered from April 24, 2025) as well as a combined chemoimmunotherapy regimen consisting of nab-paclitaxel, lenvatinib, and sintilimab (administered from May 19, 2025). Neither regimen demonstrated efficacy, as tumor markers continued to rise. Treatment was then switched on June 10, 2025, to a combination of nab-paclitaxel, sintilimab, and another FGFR inhibitor, futibatinib (an irreversible FGFR1–4 inhibitor) (20 mg once daily days 1-28, 28-day cycles). Following administration of this regimen, follow-up contrast-enhanced CT according to RECIST 1.1 showed a 6% decrease in the sum of the diameters of the target lesions relative to baseline, which still qualified as stable disease. This was accompanied by a decrease in CA19-9. However, the patient’s chest pain worsened, prompting plans for local radiotherapy. Considering the potential for increased toxicity with concurrent radiotherapy and chemotherapy, chemotherapy was discontinued. The patient was given one cycle of sintilimab on July 6, 2025, while continuing oral futibatinib, and local thoracic radiotherapy was initiated on July 8, 2025. Unfortunately, the patient died of cardiopulmonary failure on July 13, 2025, during the radiotherapy course.

**Figure 3:**
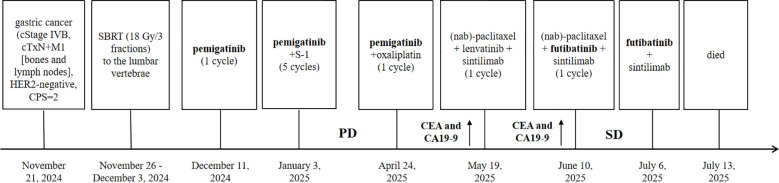
Timeline of the diagnosis and treatment of case 1.

In total, the patient received six cycles of first-line pemigatinib, achieving a progression-free survival (PFS) of 4.4 months and an overall survival (OS) of 8.3 months (**Figure 3**). As shown in **Figures 1**–**3**, after one cycle of pemigatinib monotherapy, CA19–9 and CEA levels began to decline. During five cycles of pemigatinib combined with S-1, tumor markers continued to decrease to their lowest levels (CA19-9–5 U/mL, CEA 1 ng/mL), while contrast-enhanced CT assessment showed a 2% reduction in the sum of target lesion diameters and sclerosis of bone metastases, indicating stable disease. Following completion of the five combination cycles, tumor markers gradually rose, and imaging confirmed a 24% increase in the sum of target lesion diameters, meeting criteria for progressive disease. These findings suggest initial efficacy followed by the development of acquired resistance to pemigatinib exposure.

**Figure 4:**
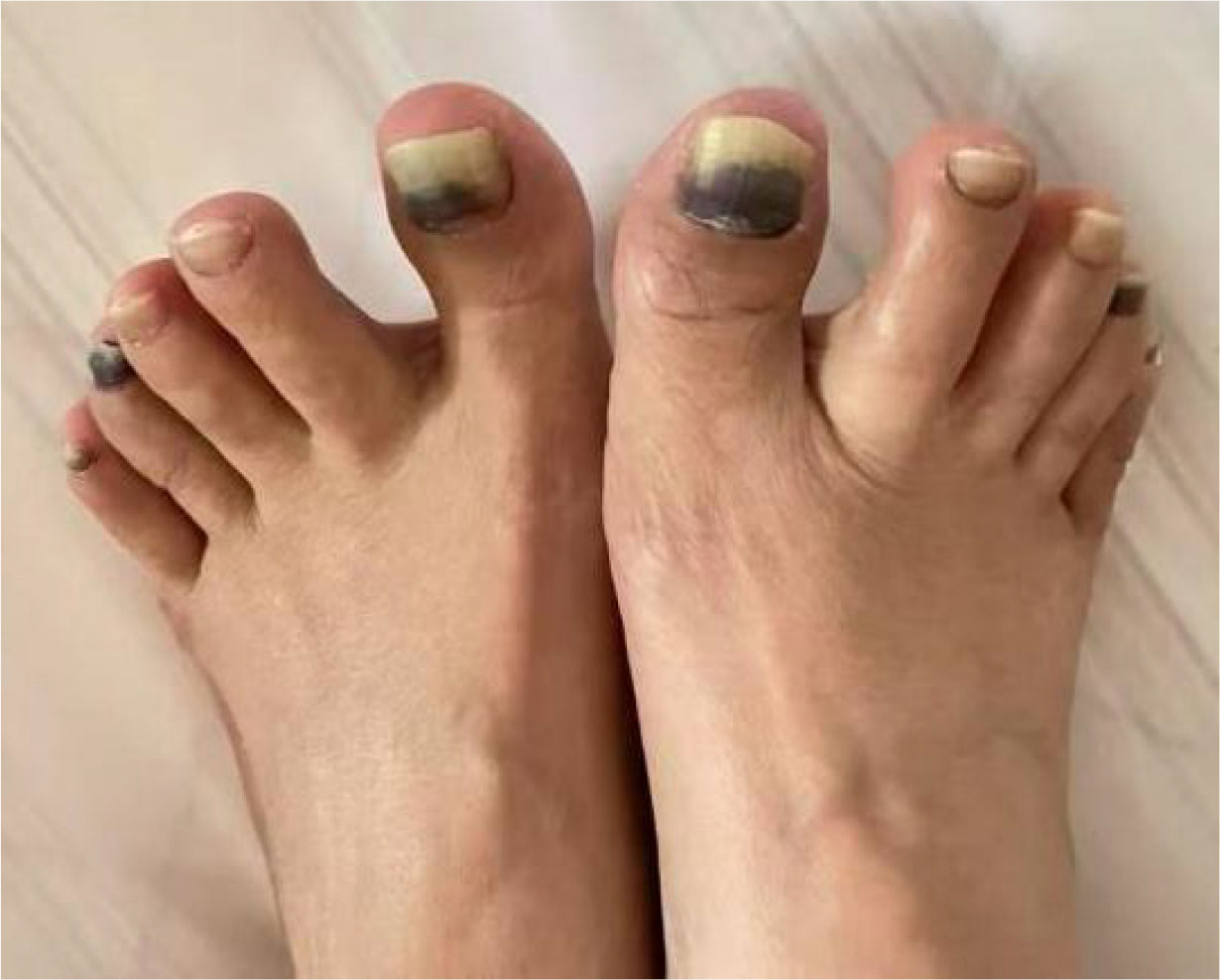
Onychotoxicity associated with pemigatinib in case 1.

During pemigatinib treatment, the patient developed grade 1–2 toxicities, including stomatitis, onychotoxicity (**Figure 4**), diarrhea, hyperphosphatemia, and palmar-plantar erythrodysesthesia. Due to intolerance to grade 2 stomatitis and palmar-plantar erythrodysesthesia, the dose of pemigatinib was reduced starting from cycle 3 to 9 mg once daily on a schedule of days 1–14 in a 21-day cycle.

### Case report 2

**Figure 5:**
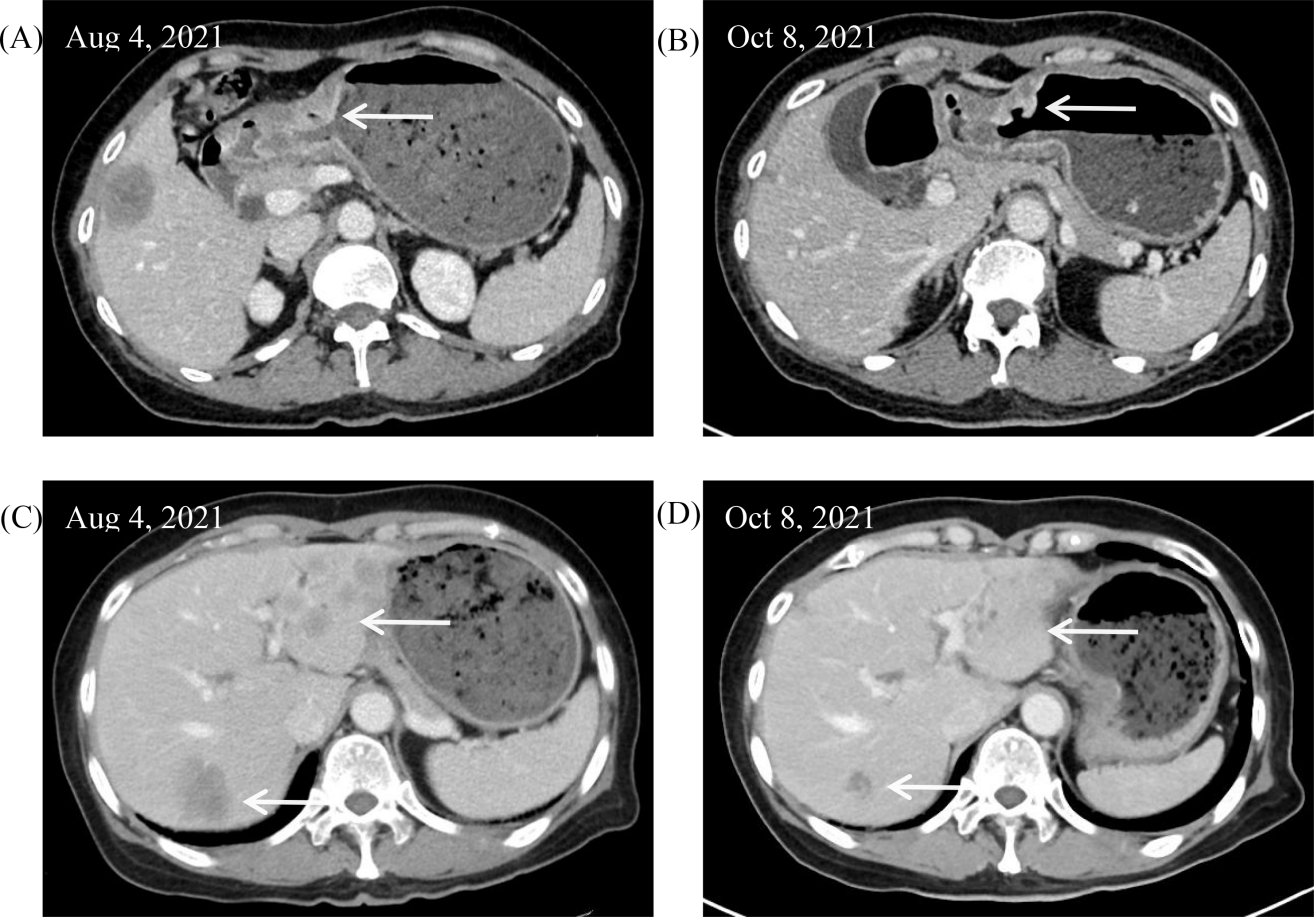
Follow-up CT scans confirming a partial response (PR) after two cycles of infigratinib. **(A, B)** Case 2 showing the gastric lesion in CT images before therapy and stable after therapy. **(C, D)** Case 2 showing marked liver metastases in CT images before therapy and a significant shrinkage after therapy.

A 61-year-old woman was diagnosed with gastric carcinoma in September 2020 [cStage IVB, cT4bN+M1 (liver and lymph nodes metastases), poorly differentiated cell carcinoma, HER2-negative, CPS = 5, microsatellite stable]. She received first-line treatment with the SOX regimen for six cycles, after which disease progression occurred. Subsequently, second-line therapy with paclitaxel was administered for four cycles, but disease progression was again observed. Concurrently, due to liver metastases, her aspartate aminotransferase (AST) and alanine aminotransferase (ALT) levels were notably elevated. Subsequently, NGS was conducted, revealing FGFR2 amplification. With appropriate consents, third-line therapy with infigratinib (inhibitors of FGFR1, 2, and 3) was started at 125 mg once daily (days 1-21, 28-day cycles). After two cycles, contrast-enhanced CT was performed to assess response according to RECIST version 1.1. The target lesions were the primary gastric lesion and liver metastases. The sum of the diameters of the target lesions decreased by 69%, confirming a partial response (PR), with significant shrinkage of the liver metastases and a stable primary lesion (**Figure 5**). Concurrently, the levels of ALT and AST decreased substantially post-therapy. The dose was reduced to 100 mg daily from cycle 3 due to grade 3 keratitis. Other toxicities included grade 1–2 palmar-plantar erythrodysesthesia, hyperphosphatemia, and xerophthalmia.

**Figure 6:**
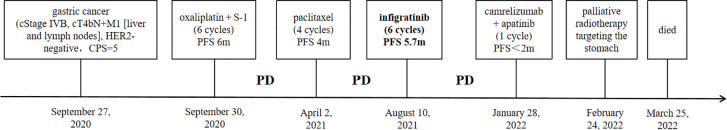
Timeline of the diagnosis and treatment of case 2.

However, disease progression occurred after six cycles of treatment, leading to the initiation of camrelizumab combined with apatinib. After one cycle, however, the patient developed pyloric obstruction, which precluded oral intake. Consequently, palliative radiotherapy targeting the stomach was initiated on February 24, 2022, with a planned dose of 45 Gy in 25 fractions. The treatment was discontinued after 21 fractions due to leukopenia and severe vomiting. The patient subsequently died from end-stage disease on March 25, 2022. This patient achieved a PFS of 5.7 months with third-line infigratinib and an OS of 18.5 months (**Figure 6**).

## Discussion

Patients with GC harboring FGFR2 amplification showed significantly worse survival, compared with patients with FGFR2-unamplified GC (12). Although diverse corresponding targeted drugs have been developed, previous studies have shown that their efficacy remains suboptimal in most patients within this subgroup (17–19). Surprisingly, this study demonstrates the clinical response of pemigatinib and infigratinib in two patients with FGFR2-amplified advanced gastric cancer. The observed PFS was 4.4 months with first-line pemigatinib and 5.7 months with third-line infigratinib, with corresponding OS of 8.3 months and 18.5 months, respectively. Both patients exhibited rapid symptomatic improvement and biomarker declines.

For Patient 1, pemigatinib provided a crucial treatment option where standard platinum/fluoropyrimidine chemotherapy was not feasible. While pemigatinib’s activity did not surpass historical first-line benchmarks in unselected HER2-negative GC (mPFS 5–7 months), its utility in this specific, treatment-limited context was clear (20). There has been scarce research reporting the efficacy of pemigatinib in advanced gastric cancer with FGFR gene alterations, with only a few case reports available, which similarly demonstrated its effectiveness (21, 22). Interestingly, after developing resistance to pemigatinib, this patient subsequently received futibatinib and still showed clinical benefit after one cycle of treatment. This suggests that irreversible FGFR inhibitors may represent a rational therapeutic strategy upon progression on prior FGFR-directed therapy. Moreover, infigratinib in the third-line in patient 2 compares favorably with historical options like nivolumab (mPFS 1.6 months) or apatinib (mPFS 2.6 months) in HER2-negative GC, highlighting its potential value in later lines (23, 24). It also demonstrated superior efficacy compared with the infigratinib monotherapy reported in Cohort 1 of the LB1001–201 trial for previously systemic second-line treated patients with locally advanced or metastatic gastric/gastroesophageal junction cancer harboring FGFR2 gene amplification, which achieved an mPFS of 3.3 months (25).

FGFR2 amplification correlated with lymph node metastasis and poorly differentiated or undifferentiated adenocarcinoma in patients with gastric cancer, consistent with our cohort’s clinical characteristics (26). The observed safety profile aligns well with toxicity data (stomatitis, onychotoxicity, diarrhea, palmar-plantar erythrodysesthesia, keratitis, hyperphosphatemia, xerophthalmia, etc.) from pivotal trials of FGFR inhibitors across tumor types, confirming predictable and manageable class effects (27, 28). This study supports the feasibility of FGFR inhibition as a viable therapeutic strategy for FGFR2-amplified advanced GC, particularly for patients who have exhausted standard therapies or are intolerant to conventional chemotherapy. Notably, clinical benefit was observed even in patients with poor performance status, a population with severely limited options. This reinforces the critical importance of NGS in identifying actionable targets like FGFR2 amplification in advanced GC to enable precision oncology approaches (29). The maintenance of efficacy in patients requiring dose reductions suggests that individualized dosing strategies, guided by tolerability and therapeutic drug monitoring where feasible, could optimize the therapeutic window for these agents.

FGFR inhibitors capable of binding to the ATP-binding site of the tyrosine kinase domain are generally classified as multikinase FGFR inhibitors (first-generation FGFR inhibitors) or selective FGFR inhibitors (second-generation FGFR inhibitors). Derazantinib, dovitinib, tasurgratinib, lenvatinib, lucitanib, nintedanib and ponatinib are all multikinase FGFR inhibitors with activity against multiple RTKs other than FGFRs. Multikinase FGFR inhibitors can potentially target both cancer cells and their microenvironment owing to the role of FGFRs and other RTKs in the regulation of angiogenesis and immunity, as well as tumorigenesis (30). However, owing to their broad activity, both the mechanisms of action and the adverse effects of multikinase FGFR inhibitors are complicated compared with those of selective FGFR inhibitors. Selective FGFR inhibitors include pan-FGFR, FGFR1/2/3 and FGFR2/3 inhibitors as well as selective FGFR2, FGFR3 or FGFR4 inhibitors (31) (**Table 1**). In gastric cancer, various FGFR-targeting small-molecule inhibitors have been evaluated. In a phase 2 study, futibatinib demonstrated modest antitumor activity in patients with heavily pretreated gastric or GEJ cancer harboring FGFR2 amplification and had a predictable and manageable safety profile (15). However, AZD4547 has been associated with limited efficacy in patients with gastric cancer. A randomized, open-label study investigated the efficacy and safety of AZD4547 monotherapy compared with paclitaxel in patients with advanced gastric adenocarcinoma harboring FGFR2 polysomy or gene amplification (18). Among all randomized patients, the mPFS was 1.8 months with AZD4547 versus 3.5 months with paclitaxel. Notably, patients with a high FGFR amplification index showed better outcomes with AZD4547 treatment (PFS 2.0 months vs. 1.4 months in those with a lower index), suggesting that the efficacy of FGFR inhibitors may correlate with the degree of FGFR amplification. Consistent with this observation, the first patient in our present report also exhibited a high amplification index (gene copy number=24.7). In addition to small-molecule inhibitors, biologic agents targeting FGF/FGFR signaling have also been developed. Among these, bemarituzumab is the only agent that is currently being evaluated in phase III trials. Bemarituzumab, a monoclonal antibody targeting FGFR2b, inhibits FGFR2 signaling on cancer cell proliferation. The phase 2 FIGHT trial demonstrated that adding bemarituzumab to chemotherapy improved survival outcomes in patients with metastatic gastric cancer and gastroesophageal junction cancer with FGFR2b overexpression (16). A novel area of recent anticancer drug development is antibody-drug conjugates (ADCs), generated by the covalent attachment of a cytotoxic drug to a monoclonal antibody via a chemical linker (32). Several innovative new approaches for targeting FGFR2 are also beginning to emerge in the preclinical setting and have been elegantly reviewed recently (33). These include proteolysis-targeting chimeras (PROTACs), chimeric antigen receptor (CAR)-T cells, selective dual inhibitor (34) and soluble receptors.

**Table 1:** Summary of anti-FGFR2 drugs.

Agents	Type	Phase (trial number)	Treatment	ORR (%)	DCR (%)	mPFS (months)	mOS (months)
AZD4547 (18)	a selective FGFR-1, 2, 3 inhibitor	phase II (NCT01457846)	AZD4547 vs. paclitaxel	2.6 vs. 23.3	N/A	1.8 vs. 3.5	N/A
infigratinib (25)	a selective FGFR-1, 2, 3 inhibitor	phase II (NCT05019794)	infigratinib	25.0	80.0	3.3	8.0
futibatinib (15)	an irreversible FGFR1–4 inhibitor	phase II (NCT04189445)	futibatinib	17.9	50.0	2.9	5.9
erdafitinib (17)	a selective pan-FGFR inhibitor	phase II (NCT04083976)	erdafitinib	13.0	63.0	N/A	N/A
bemarituzumab (16)	a humanized monoclonal antibody selective for FGFR2b	phase II (NCT03694522)	bemarituzumab plus mFOLFOX6	48.1	85.7	9.5	19.2

ORR, objective response rate; DCR, disease control rate; mPFS, median progression free survival; mOS, median overall survival; N/A, not available; FGFR, fibroblast growth factor receptor; mFOLFOX6, modified FOLFOX6 (oxaliplatin, leucovorin, 5-fluorouracil).

Preclinical data and correlative clinical observations indicate that activation of bypass signaling pathways, the emergence of secondary FGFR alterations and intratumor heterogeneity are all major causes of resistance to FGFR-targeted therapies. FGFR2 is frequently co-amplified with genes encoding other RTKs (such as EGFR, HER2, HER3 and MET) or downstream effectors (such as KRAS and PIK3CA) at distinct chromosomal loci in patients with gastric cancer (35). Two PDX models of FGFR2 and MET co-amplified gastric cancer are intrinsically resistant to FGFR-targeted therapies *in vivo* (36). FGFR2-amplified tumors can give rise to intratumor heterogeneity with copy number alterations and rearrangements in the FGFR2 gene and are able to acquire resistance to FGFR-targeted therapies (37). Gatekeeper mutations that emerge during treatment are a common mechanism of acquired resistance in FGFRs and other RTKs with primary point mutations or fusions. The FGFR2^V564F^ mutation also emerged in a PDX model of gastric cancer harboring FGFR2 amplification and overexpression during ex vivo culture with AZD4547, which caused resistance to AZD4547 and cross-resistance to infigratinib (38). Several studies have shown acquired resistance in patients taking non-covalent FGFR inhibitors such as erdafitinib and infigratinib on disease progression. Futibatinib is the only approved covalent-irreversible inhibitor reported to overcome FGFR2 mutations related to Debio 1347 or infigratinib-associated acquired resistance in patients with FGFR2 gene-altered cholangiocarcinoma (39). In this study, after disease progression following non-covalent FGFR inhibitor (pemigatinib) treatment, the patient described in Case Report 1 demonstrated a significant decrease in tumor markers and improved disease status on CT evaluation after one cycle of treatment with nab-paclitaxel combined with sintilimab and futibatinib. Although it remains unclear whether this response was primarily due to futibatinib overcoming acquired resistance or to the synergistic effect of the combination regimen, the observed efficacy suggests that covalent or extracellularly acting inhibitors, or even combination treatment with other onco-therapeutic agents, may be worth investigating for their potential to reduce FGFR-TKI resistance.

The limitations of this study are as follows. First, the sample size is extremely small (only two cases), and there is no control group or randomization. Second, response assessment was retrospective and non−blinded, and the observed progression−free survival and overall survival fall within expected ranges of variability. Third, the impact of co−occurring mutations on treatment efficacy remains unclear, and acquired resistance mechanisms were not investigated. Fourth, treatment regimens were confounded by the use of multiple agents, making it difficult to attribute efficacy to any single drug. Fifth, adverse event management reflects only individual case experience, and the dose modification strategy has not been prospectively validated. Therefore, all conclusions are hypothesis−generating observations that require validation in larger, prospective, controlled trials.

In summary, this study offers hypothesis-generating observations suggesting potential activity of FGFR inhibitors in patients with FGFR2-amplified advanced gastric cancer, particularly those with limited therapeutic alternatives and those intolerant to conventional chemotherapy. These findings underscore the therapeutic value of NGS-guided targeted therapy in this molecularly defined subset. While challenges related to access and resistance persist, our results encourage the integration of FGFR testing into routine GC management and strongly advocate for dedicated prospective clinical trials to validate these agents and optimize patient selection and treatment strategies.

## Data Availability

The original contributions presented in the study are included in the article/supplementary material. Further inquiries can be directed to the corresponding author.
